# Scientific Challenges and Implementation Barriers to Translation of Pharmacogenomics in Clinical Practice

**DOI:** 10.1155/2013/641089

**Published:** 2013-02-28

**Authors:** Y. W. Francis Lam

**Affiliations:** Department of Pharmacology, School of Medicine, University of Texas Health Science Center San Antonio, 7703 Floyd Curl Drive, San Antonio, TX 78229-3900, USA

## Abstract

The mapping of the human genome and subsequent advancements in genetic technology had provided clinicians and scientists an understanding of the genetic basis of altered drug pharmacokinetics and pharmacodynamics, as well as some examples of applying genomic data in clinical practice. This has raised the public expectation that predicting patients' responses to drug therapy is now possible in every therapeutic area, and personalized drug therapy would come sooner than later. However, debate continues among most stakeholders involved in drug development and clinical decision-making on whether pharmacogenomic biomarkers should be used in patient assessment, as well as when and in whom to use the biomarker-based diagnostic tests. Currently, most would agree that achieving the goal of personalized therapy remains years, if not decades, away. Realistic application of genomic findings and technologies in clinical practice and drug development require addressing multiple logistics and challenges that go beyond discovery of gene variants and/or completion of prospective controlled clinical trials. The goal of personalized medicine can only be achieved when all stakeholders in the field work together, with willingness to accept occasional paradigm change in their current approach.

## 1. Introduction

Variability in clinical response to standard therapeutic dosage regimen was reported in the 1950s by many pioneers in the field. Since then, the association between monogenic polymorphisms and variations of drugs' metabolism, transport, or target had been identified and the vision of personalized drug therapy in health care envisioned [1, 2]. Pharmacogenomic-guided drug therapy for patient is based on the premise that a large portion of interindividual variability in drug response (efficacy and/or toxicity) is genetically determined. Despite the widespread recognition of the scientific rationale and the clinical implementation of pharmacogenomic tests at several major academic medical institutions [3–7], most clinicians and researchers engaged in the discipline would agree that the early vision of achieving personalized therapy in the form of therapeutic regimens tailored to an individual's genetic profile remains some years away.

**Figure 1:**
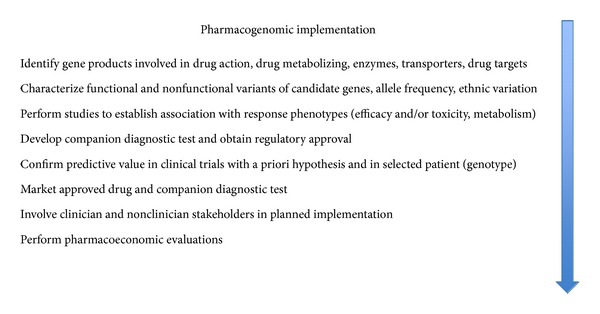
Sequence of scientific developments and implementation steps for pharmacogenomics testing in clinical practice.

Broadly speaking, the development and implementation pathways for pharmacogenomic tests consist of several stages (Figure 1): first, discovery of pharmacogenomic biomarkers and validation in well-controlled studies with independent populations; second, replication of drug-gene(s) association and demonstration of utility in at-risk patients; third, development and regulatory approval of companion-diagnostic test; fourth, assessing the clinical impact and cost-effectiveness of the pharmacogenomic biomarkers; fifth, involvement of all stakeholders in clinical implementation. Lessons learned in making pharmacogenomic-guided therapy useful to clinicians have identified multiple scientific challenges and implementation barriers existing within these stages, each of which is fueled by multitude of stakeholders with varied goals and interests [8]. This paper will provide a perspective on these existing challenges and barriers in the complex process of implementing pharmacogenomics in clinical practice, as well as incorporating pharmacogenomics into the drug development process.

## 2. Scientific Challenges and Complexity

### 2.1. Genetic Variabilities and Nongenetic Influences on Genotype-Phenotype Association

Many pharmacogenomic biomarkers have been identified over the last decade, but only few of them have been utilized to different extents in clinical setting (Table 1) [9]. One of the major challenges for translating most discovered biomarkers to their clinical implementation as genomic tests has been the inconsistent replication result of genetic associations, whether alone or in combination. Traditionally, the candidate gene approach incorporating a panel of genes that encode known drug targets, metabolizing enzymes, and membrane transporters is used in pharmacogenomic studies to test the hypothesis of an association between single nucleotide polymorphisms (SNPs) and a pharmacological or therapeutic endpoint. A good example of inconsistent replication result of genetic associations is the atypical antipsychotic clozapine with its complex pharmacological effects via the dopaminergic, serotonergic, adrenergic, and histaminergic receptors within the central nervous system. Over the years, conflicting study results exist in the literature for association between clozapine response with either SNPs of each known pharmacological receptor subtype [10–13], combinations of polymorphisms [14], and metabolizing enzymes and transporters [15]. It is also of note that the original association regarding combination of polymorphisms was not replicated in a subsequent study [16]. The recent identification of yet another new candidate gene for clozapine treatment response [15] illustrates the limitation of candidate gene approach in that there is always the possibility of involvement of other yet-to-be-identified genes, including those that have not been known to be linked to the pharmacology of the drug, that could account for additional variability in patient's therapeutic response. More importantly, the effect size of most genetic variants is small to modest. When evaluated or used alone, most of these markers are likely of insufficient sensitivity and specificity to provide clinically useful prediction, especially of efficacy.

The recognition of multiple gene variants, rather than SNPs, each accounting for part of the disposition and response phenotypes, has led to the increased use of whole genome approach for discovery of new biological pathways and identification of associations between pharmacogenomic biomarkers and response phenotypes. Genome-wide association study (GWAS) approach screens large number of SNPs (up to 2.3 million per array) across the whole genome in order to determine the most significant SNPs associated with response phenotypes. In contrast to the hypothesis-driven candidate gene approach, there is no a priori knowledge of specific gene for the discovery-driven GWAS approach. Rather, the large numbers of SNP analyses test multiple hypotheses and necessitate large sample size, sophisticated computing and platforms (e.g., Affymetrix GeneChips), and high cost. In addition, the level of significance associated with each test needs to be corrected for multiple hypothesis testings. Refinement of the GWAS approach takes a two-step design, using high-density array to discover the SNP associations in a population cohort followed by replicating the initial findings above the genome-wide significance with additional patient sets in a more hypothesis-driven study of sufficient sample size. While this approach has been successfully applied in the pharmacogenomics of clopidogrel, flucloxacillin, simvastatin, and warfarin [17–22], the implications of the results are less clear for other drugs such as the psychotropics [23–30]. 

A middle-of-the-road approach would be to limit the number of SNPs that warrant analysis. Based on the phenomenon of linkage disequilibrium among SNPs, whereby two or more SNPs are inherited together in haplotype blocks more frequently than would be expected based on chance alone [31], a single representative SNP within a haplotype block could serve as a “tag SNP” (tSNP) for the haplotype. By genotyping a smaller number of carefully chosen tSNPs to identify haplotype blocks of DNA sequences that are inherited together, researchers can capture other commonly associated SNPs within the same region. The HapMap database created by the International HapMap Project (http://www.hapmap.org/) is freely available for selection of these tSNPs. Based on the HapMap database, many GWASs of drug responses have been completed [18, 19, 32, 33]. It is hoped that some of the scientific challenges for study replication related to SNP genotyping may be alleviated through this approach [34]. 

Regardless of the choice of approach to identify the genotype-phenotype association, population variations in prevalence and relative importance of different allele variants, for example, *CYP2D6*, *HLA-B*, *UGT1A1*, and *SLC6A4*, remind investigators of the importance of ethnicity and population stratification [35, 36], which could magnify the sample size requirement for statistical power in most pharmacogenomic studies. For example, although the algorithms based on the work of Gage et al. [37] and the International Warfarin Pharmacogenetics Consortium (IWPC) [38, 39] are clinically useful, they do not include detection of the *CYP2C9∗8*, an allele commonly occurring in African Americans. The lower success with algorithm-based dose prediction in African Americans [40] is likely related to exclusion of this allele in most dosing algorithms. Another example is *HLA-B∗1502 *being a strong predictor of carbamazepine-induced severe cutaneous drug reactions in Han Chinese and most Southeast Asians but not in Caucasians, who do not carry the allele variant [41–43]. If not accounted for, these ethnicity-or population-related variables will confound the results of most pharmacogenomic association studies and could complicate the result interpretation. In addition, there is no universal agreement among different test platforms as to which allele variant should be tested routinely for some genetic polymorphisms, for example, *CYP2D6* and *UGT1A1*.

In addition to the aforementioned ethnicity-related considerations, the drug disposition and response phenotypes can be affected by patient-specific variables. Phenocopying with a change in metabolic phenotype secondary to concurrent enzyme inhibitor [44, 45] could create genotype-phenotype discordance and affect the ability to predict possible drug response based on genotype-guided dosing and achievable drug concentration. Inflammatory responses elicited by extrahepatic tumors have been shown to release cytokines such as interleukin-6 (IL-6) and resulted in transcriptional downregulation of the human *CYP3A4* gene [46]. Therefore, lower docetaxel clearance reported in cancer patients could be related to tumor-associated inflammation and subsequent transcriptional repression of *CYP3A4*, potentially leading to unanticipated toxicity despite normal enzymatic activity in the patient. IL-6-mediated downregulation of cytochrome P-450 enzyme activities also likely contributed to a recent report of significant increase in clozapine concentration in a patient with infection and inflammation [47]. An additional challenge for applying pharmacogenomic biomarkers in targeted cancer therapeutics is sampling of tumor tissue that carries the somatic mutations (e.g., testing for the epidermal growth factor receptor 1 (HER1) mutation in patients treated with gefitinib for nonsmall cell lung cancer and testing for overexpression of the human epidermal growth factor receptor 2 (HER2) protein in patients receiving trastuzumab for breast cancer). The presence of tumor cell heterogeneity might result in intra- and interindividual variabilities in tumor tissue content and, hence, measurable level of the biomarker. In spite of this limitation, there have been multiple successful clinical applications of pharmacogenomics biomarkers in selecting chemotherapeutic drugs [48].

Furthermore, there is an increasing appreciation that genetic heterogeneity alone cannot explain interindividual variations in drug responses. Yet currently, much less is known about the influence of environmental variables and gene-environment interactions on drug disposition and response phenotypes such as mutations and polymorphisms [49–51]. Epigenetics refers to changes in gene expression without nucleotide sequence alteration. Environmental factors, through their participation in epigenetic mechanisms, could result in many different phenotypes within a population. In the not too distant future, pharmacoepigenetic investigations focusing on studying the interaction among drugs, environment, and genes could provide additional insight of drug response variations beyond the level of genetic polymorphisms [52]. 

### 2.2. Analytical Validity, Clinical Validity, and Clinical Utility of Pharmacogenomic Biomarkers

After demonstration of a genetic association with response phenotype, there is the need of validating the biomarker, regardless of whether it is to be developed as a companion diagnostic test. For the purpose of personalized therapy, a companion diagnostic for a drug can be defined as a biomarker that is critical to the safe and effective use of the drug. The ACCE (analytical validity, clinical validity, clinical utility and associated ethical, legal, and social implications (ELSI)) Model Project [53] sponsored by the Office of Public Health Genomics, Centers for Disease Control and Prevention (CDC), has been recently advocated by some investigators to be the basis for evaluation of pharmacogenomic biomarker tests. Analytical validity determines how well a diagnostic test measures what it is intended to measure, regardless of whether it is an expression pattern, a mutation, or a protein. Clinical validity measures the ability of the test to differentiate between responders and nonresponders, or to identify patients who are at risk for adverse drug reactions. The clinical utility measures the ability of the test result to predict outcome in a clinical environment and the additional value over nontesting, that is, standard empirical treatment. 

In 2004, the CDC launched the Evaluation of Genomic Applications in Practice and Prevention (EGAPP) initiative, which aims to establish an evidence-based process, including assessments of analytical validity, clinical validity, and clinical utility, for evaluating genetic tests and genomic technology that are being translated from research to clinical practice. For the pharmacogenomics discipline, one often-cited publication was the 2007 EGAPP Working Group evidence-based review of the literature on the use of CYP genotyping for clinical management of depressed patients with the selective serotonin reuptake inhibitors (SSRIs). Based on strong evidence of analytical validity, possible demonstration of clinical validity, and lack of study data to support evaluation of potential clinical utility, the working group does not recommend the application of *CYP2D6* genotyping for SSRI pharmacotherapy [54].

Since approval of most CYP genotyping tests by the Food and Drug Administration (FDA) is dependent on their technical performance in detecting CYP450 gene variants, the strong evidence of analytical validity is to be expected. The weak evidence of association between genotype and phenotypes (different metabolic phenotypes, responders versus nonresponders) is also not unexpected, since most SSRIs rely on multiple but not necessarily polymorphic enzymes for metabolism and have a flat dose-response relationship with wide therapeutic index. The clinical validity of the CYP genotyping tests to differentiate response phenotypes is further limited by the CYP genotype-metabolic phenotype discordance that can occur as a result of drug-drug interactions [44, 45] or environmental influences. Given these limitations as well as the lack of cost-effectiveness data, it is not surprising that the SSRIs are not good candidates for genotype-based pharmacogenomic therapy and, hence, the recommendation of the EGAPP Working Group. Other pharmacogenomic biomarkers could be better candidates for testing association between specific genotype and clinical phenotype [55–63], as indicated by published guidelines. Pharmacogenetic dosing algorithms [37, 39] based on the patient's *CYP2C9* and *VKORC1* genotypes and other nongenetic factors (e.g., age, body size, and concurrent interacting drug) have been used to determine warfarin dosage regimens. As shown for clopidogrel, simvastatin, and warfarin, replication of the association in multiple cohorts or inclusion of replication data would provide further evidence of clinical validity [17, 18, 64, 65]. 

### 2.3. The Complexity of Defining What Constitutes Clinical Utility

Establishing the clinical utility of pharmacogenomic biomarkers has been advocated to ensure that their use is appropriate, cost-effective, and ultimately improves clinical outcome in patients. Yet within the clinical and scientific communities, there are constant debates with little agreement regarding the required levels of evidence for proof of clinical utility of diagnostic tests that are scientifically appropriate but at the same time realistically achievable [66–71]. The gold standard for demonstration of clinical utility of a drug is the use of randomized controlled trials (RCTs). Given the current evidence-based driven clinical environment, many investigators advocate that hypothesis-driven, prospective, double-blind RCTs would provide the ideal approach to validate the clinical utility of pharmacogenomic biomarkers. However, within the context of personalized medicine, the biomarker as a companion diagnostic test is intended for use with a drug to produce the optimal efficacy and safety. This makes it difficult to distinguish the clinical utility of the test that is different from that of the drug or the drug-test combination.

In addition, the traditional assessment of evidence of drug efficacy and safety with the use of RCTs may not necessarily portray the benefit of pharmacogenomic biomarkers. Complex disease etiologies, heterogeneous patient population, placebo effects, and drug response variabilities per se all contribute to statistical power issues that necessitate large patient cohort for RCT. All too often, the end result is achievement of small average benefit in the entire heterogeneous patient cohort, despite the trial being costly in terms of time and sample size. In contrast to evidence-based practice, the emphasis and value of pharmacogenomics are more geared towards incremental advantages in efficacy and safety for the outliers (the poor metabolizers, the ultra-rapid metabolizers, the nonresponders, or those susceptible to develop adverse drug reactions) over traditional therapy or standard dosing regimen. For example, the IWPC showed that a pharmacogenetic dosing algorithm was most predictive of therapeutic anticoagulation in 46% of the patients cohort who required <25 mg/week or >49 mg/week [39]. 

Therefore, a balance between the scientific demands of RCTs and the practical value of genotyping for patient care seems appropriate. Given the low prevalence of genetic variants associated with drug response and the desire to generate more robust evidence, many investigators and sponsors have advocated the use of prospective enrichment design clinical trials [72] to include patients who are more likely to respond or at least be stratified according to disease subtypes [73] and/or exclude patients who are highly susceptible to adverse drug reactions. However, even with the assumption of (and sometimes proven) association between genetic variabilities and drug response, both advantages and disadvantages exist for this study design [8]. A recent simulation study of trial designs suggested that conducting more trials with smaller sample sizes and lessened evidence-based criteria might contribute substantially to cancer survival, and assessment relying solely on the current traditional, risk-averse trial design might slow long-term progress [74]. In this regard, it is of note that the FDA recently approved crizotinib and vemurafenib with their respective pharmacogenomic biomarker tests solely on data from two single-arm studies. Finally, ethical concerns might preclude conducting RCT in patients with specific genetic polymorphisms [75]. Examples would be prescribing of abacavir in patients tested positive for HLA-*B∗5701 *and 6-mercaptopurine or azathioprine in homozygous carriers of *TMPT* mutations. Likewise, conducting a pharmacogenomic add-on as part of a head-to-head efficacy comparison of two antipsychotics in patients who are carriers of the Del allele of the −141C Ins/Del polymorphism in the dopamine D_2_ receptor gene would be difficult. The Del allele is associated with poor antipsychotic response [76]; yet, all currently marketed antipsychotics are D_2_ blocker, albeit with different extent of blockade. 

Not surprisingly, pharmaceutical companies have very little financial incentive to conduct time- and cost-intensive RCTs, especially for out-of-patent marketed drugs. To move the discipline forward to eventual implementation, we have to rethink the types of study design and/or the quality of study data for evidence of clinical validity and utility. The concept of conducting practical clinical trials in real-world setting had been previously proposed for regulatory decision-making [77, 78]. The recent study by Anderson et al. provided evidence of comparative effectiveness between pharmacogenetic-guided warfarin therapy in 504 patients versus standard care in 1,866 patients and a strong validation to the clinical benefit associated with the use of pharmacogenomic biomarkers in a real world setting [79]. At the “grassroot level,” the concept of practical clinical trial can even be modified and adopted on a much smaller scale in clinics or physician offices. As an example, elimination of tolbutamide is known to be 50% and 84% slower in carriers of *CYP2C9∗2* and *CYP2C9∗3* variants, respectively, than in homozygous carriers of *CYP2C9∗1* [80]. Yet, to-date, there is no prospective RCT to evaluate the appropriateness of 50% to 90% dose reductions for patients who are carriers of the two allelic variants. In contrast, evaluating tolbutamide efficacy can be easily done after implementation of these dosage reductions. Therefore, such effort in clinical practice, instead of expensive and time-consuming RCT, could constitute the first step of obtaining evidence of clinical utility of *CYP2C9* genotyping in optimizing tolbutamide therapy. 

For patient care, a good example for the need of balance between evidence-based medicine and personalized medicine is clopidogrel. Despite the extensive evidence of clopidogrel efficacy linked to *CYP2C19* genetic polymorphism [81, 82], debates continue over the routine use of *CYP2C19* genotyping to guide clopidogrel therapy [83–85]. This prevents more widespread use of the biomarker in individualized therapy, despite the significantly higher rates of stent thrombosis and the associated mortality rates in carriers of the reduced-function *CYP2C19∗2* allele. Based on lack of outcomes data, the joint clinical alert issued in 2010 by the American College of Cardiology and the American Heart Association did not recommend routine genotyping and suggested the need of large, prospective, controlled trials. One such trial is the Pharmacogenomics of Antiplatelet Intervention-2 (PAPI-2) trial that evaluates the effect of genotype-guided antiplatelet therapy versus standard care on cardiovascular events among 7,200 patients undergoing percutaneous coronary intervention (PCI) (clinicaltrials.gov NCT01452152). However, the results will likely not be available until 2015. The questions then become are we in the meantime sacrificing patient care on the insistence of waiting for proof of value via the evidence-based approach? If no study results are available in the near future, should we focus on steps that can facilitate the genotyping implementation in clinical setting and examine the cost-effectiveness of genotypes-guided antiplatelet therapy with a variety of different approaches? 

### 2.4. Evaluation of Cost-Effectiveness

For many healthcare facilities and systems, it is also critical to assess whether a test offers a good return on investment. Therefore, in addition to clinical validity and clinical utility, another potential barrier to test implementation is demonstration of cost-effectiveness of the companion diagnostic test. Ideally, the pharmacogenomic biomarker will result in cost-effective improved clinical care in patients who will benefit from individualized therapy with the drug and avoidance of cost-ineffective treatment for patients who likely will not benefit from the drug, either as a result of lack of response or increased adverse drug reactions [86, 87]. 

Traditional cost-effectiveness analysis compares the relative costs and outcomes of two different approaches, typically visualized on a cost-effectiveness plane divided into four quadrants [88]. As mentioned in the last paragraph, avoidance of cost-ineffective treatment is one component of cost-effective improved clinical care. Along this line, the antipsychotic drugs offer an alternative approach to cost-effectiveness evaluation for pharmacogenomics biomarkers. With an annual cost that is at least ten times higher, the atypical antipsychotic agents are more expensive yet no more efficacious and, hence, likely to be less cost-effective, than the typical antipsychotic agents [89, 90]. Rather than focusing on using biomarkers to predict efficacy of the more expensive atypical antipsychotic agents [10–15], genotyping for the *Glycine9* allele of the *Ser9Gly* polymorphism in the dopamine 3 receptor gene [91, 92] might be used to identify patients susceptible to tardive dyskinesia, a highly prevalent adverse drug reaction associated with the use of the less expensive typical antipsychotic agents. The genetic testing might enable appropriate dose reduction for the typical antipsychotic agents and lessen the incidence of adverse drug reaction. 

Additional approaches of demonstrating cost-effectiveness of pharmacogenomic-based therapy can range from clinical trial comparing per-patient cost for specific clinical outcome between genotype-based regimen and standard regimen [93] to decision model-based study using simulated patient cohort [94–96]. Alternative approach exists even within the context of cost-effectiveness comparison between genotype-based regimen and standard regimen with no genetic testing. With generic availability of clopidogrel, a cost-effectiveness study of the value of pharmacogenomic biomarker should compare clopidogrel use in *CYP2C19* EMs and UMs versus the use of prasugrel or ticagrelor for PMs. 

Regardless of the specific approach, it should be understood that the economic impact and cost-effectiveness of screening could be affected by different variables. Two separate studies utilized modeling techniques with simulated patient cohorts to evaluate the potential clinical and economic outcomes for pharmacogenomic-guided warfarin dosing. While the relatively high cost of *CYP2C9* and *VKORC1* bundled test ($326 to $570) resulted in only modest improvements (quality-adjusted life years, survival rates, and total adverse rates), the investigators also suggested that improvements in the cost-effectiveness can be achieved in several ways, specifically further cost reduction of the genotyping test and utilizing genotype-guided warfarin dosing algorithm in outliers (patients with out-of-range INRs and/or those who are at high risk for hemorrhage [97, 98]). The benefits of pharmacogenomics-guided therapy for patient subpopulations have been discussed earlier. Other variables such as different population prevalence of a specific variant and cost of alternative treatment approaches would also impact the economic impact analysis. 

In summary, clinical utility and cost-effectiveness cannot be the only measures in determining the relative value of pharmacogenomics for drug therapy optimization in individual patients. Rather, they should be used to supplement the best practice strategies currently in place to achieve optimal drug therapy. 

### 2.5. Regulatory Approval of Pharmacogenomic Diagnostic Tests

Over the last decade, the FDA has progressively acknowledged the importance of biomarkers and provided new recommendations on pharmacogenomic diagnostic tests and data submission. These efforts included the publication of FDA Guidance for Pharmacogenomic Data Submission, Guidance on Pharmacogenetic Tests and Genetic Tests for Heritable Markers, and draft guidance for “In Vitro Diagnostic Multivariate Index Assays” (IVDMIAs), the introduction of the Voluntary Data Submission Program, and formation of an Interdisciplinary Pharmacogenomic Review Group (IPRG) to evaluate the voluntary submissions, as well as the approval and classification of different biomarkers [99]. Obviously, any biomarker with FDA approval will generate more confidence for clinicians, healthcare facility administrators, and payers, and could enhance test implementation and utilization in the clinical settings. Additional regulatory efforts also provide an impetus of pharmacogenomic data submission for drug approval and additional research to address the debate over the utility of the information incorporated in the revised labels, for example, for clopidogrel [83–85].

Within the United States, there are separate regulatory oversights for a pharmacogenomic biomarker developed as an in-house test by a clinical laboratory versus that for an in vitro diagnostic device developed by a medical device manufacturer. Quality standards for clinical laboratory tests are governed by the Clinical Laboratory Improvement Amendments (CLIA). In addition, the laboratories are accredited either by the College of American Pathologists, the Joint Commission on Accreditation of Healthcare Organizations, or Health Department of each individual state, that take into consideration of CLIA compliance and laboratory standard practices that are in line with Good Laboratory Practice (GLP) regulations enforced by the FDA. Although there is internal validation within the laboratory, there is no external regulatory review process for the test itself. 

 On the other hand, the GLP regulations govern the testing of in vitro medical diagnostic device. Although currently there is no formal regulatory process for submission of companion diagnostic tests, the FDA previously ruled that evaluation and approval of the AmpliChip CYP450 Test as an in vitro diagnostic device was required. In addition, the regulatory agency had fast track approved trastuzumab with the companion diagnostic Hercep Test in 2001 for detecting overexpression of HER2 protein in breast cancer tissue by immunohistochemistry and more recently for tests that utilize fluorescence in situ hybridization to amplify the *HER2* gene. Further examples of FDA assuming a greater role were the respective companion diagnostic tests approved for crizotinib and vemurafenib. With the formation of a personalized medicine group within the Office of In Vitro Diagnostic Device, Center for Device Evaluation and radiological Health, it is likely that more FDA-approved tests would be available in the future [100]. Although no similar frameworks for premarketing regulatory review and approval of pharmacogenomic biomarkers exist in the European Union and the United Kingdom, there are regulations applicable for postmarketing approval. Gefitinib was approved by the European Medicines Agency (EMA) in June 2009, followed by subsequent approval of a companion diagnostic test for *HER1* mutations. 

## 3. Integration of Pharmacogenomic Biomarker within the Healthcare System

There are several challenges and practical aspects related to clinical decision support infrastructure and training of healthcare professionals (Table 2) that need to be addressed before pharmacogenomic biomarkers can be successfully utilized in any healthcare setting. These are further discussed in the following sections.

### 3.1. The Multifacet Process of Clinical Implementation

Even with a decrease in genotyping cost over time, a relatively low demand for specific biomarker test at institutional clinical laboratories may not justify the cost of equipment and technical upkeep associated with in-house testing. This not only precludes the ideal point-of-care consultation at the bedside or within the clinic, but also results in long turnaround time for obtaining test results from external clinical laboratories or research institutions. The impact of the time delay would depend on the “urgency” of the test, for example, HER2 expression or *CYP2C19* genotyping prior to scheduled PCI versus on-the-spot warfarin dosing adjustment or in the setting of emergency PCI. Nevertheless, progress has been made in this aspect. A recent commentary of pharmacogenomics in primary care reported acceptable turnaround time of 24 hours for a feasibility study of warfarin pharmacogenetic testing in a family practice clinic [101]. In addition, a point-of-care *CYP2C19* genotyping device with a turnaround time of about an hour has been developed and recently used to explore the feasibility of incorporating *CYP2C19∗2* testing into clinical protocol for antiplatelet dosing [102]. In addition to technology advances, the concept and adoption of preemptive (preprescription) genotyping [5, 103–105] with result stored in electronic medical record for subsequent use would also help minimize the inconvenience of time delay in test reporting. The issue of health informatics technology will be discussed in a later section. 

Not unexpectedly, patients expect healthcare professionals to be able to explain the pharmacogenomic diagnostic test results and answer their questions regarding treatment access and choices. While interpretation of genotype result for deciding the appropriateness of a specific drug for a patient is usually not difficult, for example, the presence of the *HLA-B∗5701* variant for excluding abacavir therapy in patients with HIV-1 infection, the contrary would be true when the genotype result is used for dosing adjustment. The challenges for genotype-based doing guidelines [106] are related to the multitude of genetic and nongenetic variables that can affect drug disposition and response, the significant interindividual variabilities in activities of most of the metabolizing enzymes, and the possibility of phenocopying with metabolic phenotype change in the presence of drug-drug interaction [44, 45]. This difference in interpretation complexity related to the intended use of the test is likely one of the reasons for the FDA to previously separate pharmacogenomic biomarkers into three categories. Despite these challenges, warfarin dosing recommendations based on *CYP2C9* and *VKORC1* genotypes have been incorporated by the FDA into the updated product label in 2010. The dose table provided in the product label was reported to provide better dose prediction than empiric dosing [99, 107]. 

However, the inclusion of most of the pharmacogenomics biomarkers as informational pharmacogenetic tests by the FDA on the revised labels of many drugs, without clear guidance on dosing recommendation and/or therapeutic alternatives, usually results in a “knowledge vacuum” for the clinicians. All stakeholders would agree that lack of sufficient pharmacogenomics education for health professionals remains a major barrier for practical implementation of pharmacogenomics within the healthcare system [8]. The need of adequate training was echoed in a recent USA survey of more than 10,000 physicians. Although 98% of all respondents agreed that the genetic profile of a patient could influence drug therapy decision, only 29% had received some pharmacogenomics education during their medical training, and only 10% felt they were adequately trained to apply the knowledge in clinical practice [108]. Although the International Society for Pharmacogenomics recommended incorporating pharmacogenomics education in medical, pharmacy, and health science curricula [109], pharmacogenomics courses or materials have only been included to a variable extent at most pharmacy schools [110, 111]. The gap in knowledge can currently be addressed through clinical guidelines available from professional organizations (Clinical Pharmacogenetics Implementation Consortium, the International AIDS Society-USA panel, the European Science Foundation, the British Association of Dermatology, and the Pharmacogenomics Working Group of the Royal Dutch Association for the Advancement of Pharmacy) [55–63, 112], availability of simple dosing algorithm such as that for warfarin [79], and further effort to include specific dosing recommendation in product label [99, 107]. 

The most logical setting for initial implementation of pharmacogenomics would be healthcare facilities affiliated with academic institutions. The concept of pharmacogenomics-guided drug therapy is similar to that of clinical pharmacokinetics consultation service (CPCS) or therapeutic drug monitoring (TDM) program. In this regard, the familiarity of the CPCS or TDM program should be emphasized to clinicians who view the adoption of pharmacogenomics with some skepticism. Likewise, hospitals with established CPCS or TDM program might find the task of introducing pharmacogenetic testing less formidable simply by expanding or modifying their existing clinical services. The availability of consultation service, in any format, should be complemented by educational training of clinicians to achieve specific competences. Crews et al. reported significant increase in ordering of the *CYP2D6* genotyping test one year after its availability via the CPCS [3]. In a similar manner, once more clinicians are educated about the utility of pharmacogenomic approach to drug therapy, especially how to use the information, they would over time integrate pharmacogenomic findings and technologies into their practice. 

The importance of healthcare informatics for implementation of pharmacogenomics in clinical practice could not be overemphasized. At the level of patient care, integration of genotyping order template and/or genotype result into a robust system of electronic medical record (EMR) with pop-up action alert and order templates for actionable pharmacogenomic tests to be used by physicians will be necessary [113, 114]. At the level of research, the health information technology would enable organizational management of all research data and accessibility by the EMR [115–118]. Both the patient care- and research-level informatics should incorporate updated information when available and be linked to other health informatics such as billing, clinical laboratory, and clinical trials within the healthcare facility. Although adoption of EMR is not universal [119], health information technology is a critical area for investment by healthcare system administrators, perhaps through collaborative efforts with the technology industry and the government. Successful examples incorporating a coordinated team approach (physicians, pharmacists, information technology and laboratory personnels) with appropriate infrastructure support (informatics) to facilitate clinical implementation of pharmacogenomics have been reported at several institutions [3–5]. 

To fully integrate the multifacet process of the pharmacogenomics service, other organizational aspects of clinical decision support should include fostering effective communication and collaboration between laboratory staff and clinicians, creating flexible workflow with minimal disruption to the daily activities of the practitioners, delineating policies and reward systems that allow equitable schedule to minimize the additional “time burdens” perceived by some healthcare providers, and standardizing procedures to incorporate up-to-date pharmacogenomics-related information into formulary review and decision by the pharmacy and therapeutics committee. All these steps would facilitate implementation with minimal effect on work efficiency and cost for the healthcare system.

### 3.2. Reimbursement Issues

Successful implementation of pharmacogenomic biomarkers in clinical practice not only involves multidisciplinary coordination among physicians, pharmacists, clinical laboratories, health information specialists, and healthcare system administrators, but also requires collaborative efforts and willingness from the payer, a significant stakeholder in this endeavor. With the current healthcare landscape and the high cost of providing healthcare, the reimbursability of any particular test plays a significant role in deciding its implementation status in most healthcare facilities. While the cost of testing for several oncologic biomarkers and thiopurine S-methyltransferase in the United States is reimbursed in some hospitals, that is not the case for most pharmacogenomic biomarker tests. Both federal and private payers are reluctant to reimburse the cost of the tests on the basis of either (1) lack of evidence of clinical utility (which is usually associated with endorsement by professional organizations), (2) tests being not medically necessary (because it has never been classified by the FDA as required test), or (3) lack of cost-effectiveness analysis and/or comprehensive comparative effectiveness analysis. Even with the product labeling information regarding the impact of CYP variants for warfarin, the Centers for Medicare and Medicaid Services recently denied coverage for genetic testing except when the test is provided for the purpose of clinical trials. This reluctance stance is consistent with the findings by Cohen et al. [120] who reported that most payers do not consider test accuracy in identifying subpopulations of interest, test cost, medication adherence, and off-label use as relevant factors in their consideration for reimbursement. In their survey of 12 payers, the most consistent determining factor is conclusive evidence linking the use of the diagnostic test with health outcome. 

Even though most payers understand the implications of pharmacogenomics in healthcare and the potential return on investment, their reluctance to pay for diagnostic tests costing much less (most costing ≤ $500) than what they actually pay for the more expensive drugs (for which the diagnostic tests could be useful) primarily reflects their expectation of demonstration of clinical utility and comparative effectiveness [120, 121]. Accordingly, inconsistent assessment of clinical utility and benefit could only result in confusion regarding the appropriate use and interpretation of biomarker-based pharmacogenomic diagnostic tests. Hopefully, more realistic clinical practice guidelines from diverse groups of organizations and expert panels that take into consideration of the issues discussed earlier, would pave the way to greater extent of implementation. To that end, it is of note that regulatory guidance [122] has been published to support the recommendation of the clinical practice guidelines. In addition, additional clarification from regulatory agencies regarding definition of clinical utility, especially in the context of distinguishing the difference between utility of a diagnostic test versus test/drug combination versus the drug itself, would be very helpful in dealing with issues of implementation decision and test reimbursement. 

It should also be noted that even for trastuzumab, which is reimbursed by most insurers, there have been few cost-effectiveness analysis of HER2 protein expression and treatment with trastuzumab [123]. For most pharmacogenomic biomarkers, the ideal analyses might not be available until years after the diagnostic test is marketed. With limited comprehensive pharmacoeconomic data for cost-effectiveness evaluations [124, 125], other evaluation approaches ranging from comparing per-patient cost for specific clinical outcome within in-patient setting [93] to decision model-based study that utilizes simulated patient cohort [94–96] should be considered. In addition, all stakeholders should recognize that a “negative” cost-effectiveness conclusion based primarily on high cost of genotyping needs to be interpreted with the high likelihood of lower cost of genotyping in the foreseeable future. 

Since revenue generation from a pharmacogenomic diagnostic companion test would likely be significantly less than that for a drug, there is not much incentive for pharmaceutical companies to include a thorough cost-effectiveness analysis as part of drug development. With much less financial resources than pharmaceutical companies, the lack of incentive for conducting similar evaluations also applies to diagnostic companies developing the biomarkers. In a way similar to the mutually beneficial codevelopment of proprietary drug and diagnostic test [126, 127], one possible solution is for diagnostic companies to collaborate with other stakeholders, such as pharmacy benefit manager (PBM), to generate the evidence deemed necessary for reimbursement by both private payers and regulatory agencies. Medco is the first PBM to use claims data in demonstrating a 28% reduction in bleeding or thromboembolic events in patients whose physicians were provided with *CYP2C9* and *VKORC1* genotypes results, when compared to patients without genetic testing. Concurrent with the clinical effectiveness data is a $910 cost saving over a 6-month study period in the genotyped group [128]. This type of economic impact data for pharmacogenomic testing could be used as evidence of cost-effectiveness to insurance payers and administrators of healthcare systems for consideration of potential implementation.

Given the dilemma of insistence of evidence-based data for reimbursement and the limited financial resource of most diagnostic companies in developing the biomarker, some paradigm shifts in thinking about approaches to reimbursement decision could be offered to the payers. Instead of a universal reimbursement for all patients tested for a pharmacogenomic biomarker, an action-based reimbursement could be instituted. Using clopidogrel as an example, the differential reimbursement could take the form of no payment for the *CYP2C19* genotype test, if no PCI is performed and clopidogrel is not prescribed, or even different amount of payment based on the risk of PCI. This differential pay concept is currently in place for most prescription drugs in the form of copayment, as well as in coverage amount between within-network versus out-of-network physician visits [110]. Adopting such approach would lessen the financial burden for payer since the cost of the one-time test could be easily covered through cost saving associated with not using the drug when it is ineffective or harmful in specific patient populations, and it could provide a work-around to some payers' insisting on conclusive evidence of linking diagnostic tests to health outcomes [120].

### 3.3. Ethical, Legal, and Social Issues

Implementation of pharmacogenomic testing could result in situations where an individual's disease or medical condition is revealed to other parties, however unintended, as well as potential for discrimination and ineligibility for employment and insurance. Therefore, even though the public is in general receptive to genetic-based prescribing [129, 130], effort should be directed towards alleviating their concern regarding privacy and confidentiality for the purposes of employment and insurance coverage decisions. They should be informed that there are ways to both protect patients' privacy whilst at the same time promote the pharmacogenomic implementation in clinical practice [131, 132]. In addition, provisions from the 2008 Genetic Information Nondiscrimination Act were designed to protect individuals from genetic discrimination. Addressing these concerns also encourages informed patients to participate in necessary research [115, 133], for example, comparative effectiveness requested by other stakeholders, as well as facilitate healthcare professionals' willingness to fully integrate genomic services into clinical practice. Despite this, other existing concerns include ownership of genetic materials, availability and access to the information (both locally and across different health system facilities similar to that of the Veterans Affairs EMR), and patient's awareness of the consequences of storing genetic materials and phenotypic data. These concerns would need to be addressed to the satisfaction of all stakeholders, especially the patients.

Most discussions and debates on the ethical, legal, and social implications of genetic tests usually make few distinctions between pharmacogenomic biomarkers designed for drug therapy individualization and genetic tests predicting disease susceptibility that usually carry a much greater potential for abuse. For the purpose of implementation, it would seem appropriate that consent for pharmacogenomic biomarker tests designed to individualize their drug therapy (choice and/or dosage regimen) not be treated the same extent of scrutiny and requirement as genetic testing for disease susceptibility. A lessening in regulation and consent requirements for pharmacogenomic markers might make it easier for their implementation. However, this issue of is very much open for further discussion before consensus can be made.

Social concerns also arise from clinical implementation of pharmacogenomic biomarkers within the healthcare systems. In the United States, patients are required to pay for some of the cost of the medical service, either in the form of copayment or coinsurance. Therefore, an individual patient's socioeconomic status could preclude any potential beneficial pharmacogenomic test information and exacerbate health-care disparities among different patients. In addition, for patients who are identified by pharmacogenomic test either as nonresponders or at high risk of adverse drug reaction to a specific drug, the use of pharmacogenomic test as a “gatekeeper” of accessibility to drug treatment might pose a problem if there is no suitable alternative drug available. As discussed earlier in this paper, carriers of the Del allele of the 141C Ind/Del polymorphism of the dopamine D_2_ receptor gene are predicted to have poor response to antipsychotic treatment; yet, all currently marketed antipsychotic treatments possess D_2_ blockade. How then should those patients be advised and treated? Is it ethical or appropriate if the patient and/or the physician decide to use a drug regardless of the unfavorable response and/or risk associated with a specific genotype? These are relevant questions since the clinical validity and clinical utility of most pharmacogenomic tests have not been universally accepted in clinical practice. Another potential concern is liability for the healthcare provider. If a pharmacogenetic test (e.g., *CYP2C29*) is used to guide therapy with one drug (e.g., warfarin) and the patient is later prescribed another drug that is also affected by the gene previously tested (e.g., phenytoin), should the clinician be responsible to act on the genotype results when dosing the second drug? If the answer is affirmative, then some point-of-care mechanism must be in place, for example, in an EMR with pop-up action alert containing the pharmacogenomic information, so that the clinician is aware of genetic test results relevant to the prescribed drug. The immediate implication with availability of pharmacogenomic information within the EMR is that the information should not be ignored for clinical, ethical, and legal reasons.

Pharmacogenomic biomarker tests are a subset of the increasing universe of genetic tests advertised over the internet directly to the consumer. Most of these direct-to-consumer (DTC) genetic tests are “home brew” and not subject to regulatory oversight by the FDA and/or CLIA compliance for test quality standards and proficiency. In addition, companies selling DTC genetic tests can develop and market them without establishing clinical utility, which contrasts significantly to that demanded for pharmacogenomic biomarkers discussed earlier in this paper. The lack of regulatory oversight and concern of test validity likely contribute to the conclusion that most DTC genetic tests are not useful in predicting disease risk [134, 135]. Current knowledge suggests that genomic profiling based on a single SNP, a common feature to most DTC genetic tests, is not necessarily clinically accurate or useful. In this regard, the recent report of a DTC genome-wide platform [136] could provide a useful example of the impact of pharmacogenomic profiling on patient care. Despite the increased consumer desire for health-related information and personalized medicine, most patients would need the help of clinicians to differentiate the relevance of different pharmacogenomics tests. This underscores the importance of educating clinicians and preparing them to provide the appropriate test interpretation for clinical decision-making. 

## 4. Incorporating Pharmacogenomics into Drug Development

Incorporating pharmacogenomics into the entire drug development process holds significant potentials for more efficient and effective clinical trials as well as financial implications for the industry. However, the issues of sufficient sample size, the cost and time associated with conducting a RCT to address a specific study hypothesis, and the logistics of ensuring privacy concerns of institutional review board with possible delay in study approval and subject enrollment have posted a significant challenge and deterrent for the industry to fully incorporate pharmacogenomics in different phases of drug development [137]. In addition, the blockbuster drug concept and its financial impact on revenue have historically played a major role in pharmaceutical drug development. As such, the concept of pharmacogenomics and the resultant segmented (and smaller) market tailored to a subpopulation with specific genotype have been viewed unfavorably because of lower revenue and decreased profit. However, trastuzumab provides a good example of the benefit of paradigm shift in thinking about market share and revenue. The manufacturer's development of trastuzumab along with the diagnostic device results in capturing the market share associated with breast cancer drug treatment in all, albeit at a smaller number, of the women overexpressing the HER2 protein. 

There are additional drug development advantages associated with this “mental shift” in business model from the traditional approach of product differentiation to the new commerce of market segmentation, sometimes even with little or no competition. Identifying patients likely to respond to participate in clinical trials could enable benefits to be shown in a smaller number of patients, resulting in more efficient phases II and III studies conducted in shorter time frame and reducing the overall cost of drug development. It could also screen out patients likely to have unfavorable side effects that only appear in phase IV postmarketing surveillance studies, and such undesirable events sometime could lead to the inevitable and unfavorable outcomes of postmarketing product recall and litigation. The litigation and financial burden could be further minimized if the pharmaceutical company works with regulatory agencies to incorporate the pharmacogenomic information into a drug label that more accurately describes contraindications, precautions, and warnings [138]. Finally, as indicated earlier in this paper, beneficial partnership to develop and market a companion diagnostic test can also lead to additional revenue stream [127].

With more than 50% of new chemical entities failing in expensive phase III clinical trials, high attrition rate in drug development is a well-known fact for the pharmaceutical industry, and a much less discussed and explored role of pharmacogenomics is the potential of “rescuing” drugs that fail clinical trials during drug development. The prime example for this benefit is gefitinib, which originally was destined to failure because only a small number of patients with small cell lung cancer responded to the drug. However, in 2004, published results showed that tumor response to the drug was linked to mutations in *HER1*. Subsequently, development of pharmacogenomic biomarker tests for *HER1* mutations in patients enables identification of responders for gefitinib [139–143]. This example showed that investigational drugs found to be ineffective or unsafe during phase II or III clinical trials might deserve a second look from the perspective of pharmacogenomics. Another example is lumiracoxib, a selective cyclooxygenase-2 inhibitor that was withdrawn in 2005 from most global pharmaceutical markets because of hepatotoxicity. Recently, Singer et al. reported a strong association between patients with *HLA-DQ* variant alleles, especially *HLA-DQA1∗0102,* and elevated transferase levels secondary to lumiracoxib-related liver injury [144, 145]. As a result, the manufacturer of lumiracoxib has submitted an application to the EMA for its use in targeted subpopulations. 

Therefore, as demonstrated by gefitinib and possibly lumiracoxib, “failing” drugs can be further developed with a smaller target population with the genetic profile predictive of improved efficacy and/or reduced toxicity. This result can then be used for approval with appropriate product label containing the pharmacogenomic information. In reality, a go-ahead decision by the pharmaceutical company for such “drug rescue” with potential drug approval is dependent not only on the cost and time associated with developing a companion diagnostic test but also measurable better efficacy than competitor drugs in a smaller number of patients. To facilitate this aspect of drug development, regulatory “decision incentives” in the form of conditional approval with subsequent requirement of phase IV trial or approval similar to those developed and submitted under the Orphan Drug Act could go a long way to provide sufficient incentive for the pharmaceutical industry.

Regulatory agencies worldwide, primarily the FDA, the EMA, and the Japanese Pharmaceuticals and Medical Devices Agency, have recognized the opportunity to utilize pharmacogenomics in predicting drug response and incorporated pharmacogenomic information into revised labels of approved drugs as well as regulatory review, for example, by the IPRG of the FDA, that is independent of the drug review itself. Nevertheless, relevant drug efficacy and safety data and issues that are important for regulatory decision-making were developed long before the era of pharmacogenomics, and it is unclear how traditional regulatory review would approach the inclusion of any pharmacogenomic data in a new drug application (NDA) package. As described earlier, the FDA has developed multidisciplinary workshop [146] as well as regulatory initiatives such as the Voluntary Exploratory Data Submission in the USA, and the Pharmacogenomics Briefing Meetings in Europe and Japan have attempted to encourage the use and submission of pharmacogenomic data by the pharmaceutical industry. However, concerns and questions remain regarding what type of pharmacogenomic data is necessary and when they should be incorporated in the NDA process [8]. 

## 5. Conclusion

Although significant scientific and technological advances enable identification of variants in (or haplotypes linked to) genes that regulate the disposition and target pathways of drugs, translating the pharmacogenomic findings into clinical practice has been met with continued scientific debates, as well as commercial, economical, educational, ethical, legal, and societal barriers. Despite the well-known potentials of improving drug efficacy and safety, as well as the efficiency of the drug development process, the logistical issues and challenges identified for incorporating pharmacogenomics into clinical practice and drug development could only be addressed with all stakeholders in the field working together and occasionally accepting a paradigm change in their current approach.

## Figures and Tables

**Table 1:** Selected examples of drugs with relevant pharmacogenomic biomarkers and context of use.

Drugs	Pharmacogenomic biomarker or variant allele	Response phenotype	Regulatory decision and/or clinical recommendation
Abacavir	*HLA-B*∗5701	Hypersensitivity reactions	FDA and EMA warn of increased risk in patients with *HLA-B*∗5701. Genetic screening recommended before starting therapy. Patients tested positive should not receive abacavir.
Azathioprine and 6-mercaptopurine	Defective *TPMT* alleles (e.g. *TMPT*∗*2*)	Myelosuppression	Increased risk for myelotoxicity in homozygotes treated with conventional doses. FDA recommends genetic testing prior to treatment.
Carbamazepine	*HLA-B*∗1502	Stevens-Johnson syndrome (SJS) and toxic epidermal necrolysis (TEN)	FDA warns of increased risk for increased risk of SJS and TEN in patients with *HLA-B*∗1502. Patients from high-risk regions (e.g., Southeast Asia) should be screened for *HLA-B*∗1502 before starting carbamazepine.
Cetuximab and panitumumab	*EGRF, KRAS *	Efficacy	With clinical benefit limited to patients with EGRF-positive tumors, both chemotherapeutic drugs are indicated for EGRF-expressing colorectal cancer with wild-type *KRAS*. They may be ineffective in patients with tumors expressing *KRAS* mutation. Mandatory testing required.
Codeine	Duplicated or amplified *CYP2D6* alleles	CNS depression	FDA warning regarding patients who are ultrarapid metabolizers secondary to the *CYP2D6*∗*2XN* genotype would have much higher morphine concentration, and at increased risk for CNS symptoms related to overdose, even when treated with standard doses.
Clopidogrel	Defective *CYP2C19* alleles (e.g. *CYP2C19*∗*2*, *CYP2C19*∗*3*)	Efficacy	FDA warns of possible reduced effectiveness in *CYP2C19* homozygotes.
Crizotinib	*ALK *	Efficacy	Mandatory testing required by the FDA to confirm the presence of lymphoma kinase (ALK) mutation prior to drug use.
Gefitinib	*EGRF *	Efficacy	Approved by EMA for treatment of EGRF-expressing tumors.
Imatinib	*BCR-ABL* translocation	Efficacy	Mandatory testing required by the FDA for confirmation of disease and selection of patients for which the drug is indicated.
Irinotecan	*UGT1A1*∗*28 *	Neutropenia	FDA recommends dosage reduction by one level in homozygotes.
Maraviroc	*CCR-5 *	Efficacy	FDA and EMA approved indication is only for HIV infection with CCR-5-tropic-HIV-1.
Trastuzumab	*HER2 *	Efficacy	FDA and EMA require mandatory testing for HER2-overexpressing cancers prior to treatment.
Vemurafenib	*BRAF* V600E mutation	Efficacy	FDA requires mandatory testing for the mutation prior to drug use.
Warfarin	*CYP2C9* *VKORC1 *	Efficacy and toxicity (bleeding)	FDA provides dose recommendations according to *CYP2C9* and *VKORC1* genotypes.

**Table 2:** Practical issues involved in clinical implementation of pharmacogenomic testing in healthcare system.

Issue	Challenge
Test performance	Reasonable turnaround time for delivery of test result
Interpretation of result	Not a straightforward normal versus abnormal interpretation Education of clinicians is crucial to proper use
Education of health professionals	Variable time and content devoted to educating future clinicians within health professional schools Overwhelming information for most current practicing clinicians
Cost reimbursement by payers	Almost exclusively based on proof of cost-effectiveness
Acceptance by clinicians	Potential additional workload Potential legal liability Health disparity concern for patient
Acceptance by patients	Privacy and discrimination concern Health disparity concern Ownership of genetic information

## References

[1] Evans WE, Relling MV (1999). Pharmacogenomics: translating functional genomics into rational therapeutics. *Science*.

[2] Vesell ES (1984). New directions in pharmacogenetics: introduction. *Federation Proceedings*.

[3] Crews KR, Cross SJ, Mccormick JN (2011). Development and implementation of a pharmacist-managed clinical pharmacogenetics service. *American Journal of Health-System Pharmacy*.

[4] Nelson DR, Conlon M, Baralt C, Johnson JA, Clare-Salzler MJ, Rawley-Payne M (2011). University of florida clinical and translational science institute: transformation and translation in personalized medicine. *Clinical and Translational Science*.

[5] Pulley JM, Denny JC, Peterson JF (2012). Operational implementation of prospective genotyping for personalized medicine: the design of the vanderbilt PREDICT project. *Clinical Pharmacology and Therapeutics*.

[6] O'Donnell PH, Bush A, Spitz J (2012). The 1200 patients project: creating a new medical model system for clinical implementation of pharmacogenomics. *Clinical Pharmacology and Therapeutics*.

[7] Cavallari L, Nutescu EA (2012). A team approach to warfarin pharmacogenetics. *Pharmacy Practice Nerws*.

[8] Lam YWF, Lam YWF, Cavallari LH (2013). Translating pharmacogenomic research to therapeutic potentials. *Pharmacogenomics: Challenges and Opportunities in Therapeutic Implementation*.

[9] Zineh I, Pebanco GD, Aquilante CL (2006). Discordance between availability of pharmacogenetics studies and pharmacogenetics-based prescribing information for the top 200 drugs. *The Annals of Pharmacotherapy*.

[10] Malhotra AK, Goldman D, Buchanan RW (1998). The dopamine D3 receptor (DRD3) Ser9Gly polymorphism and schizophrenia: a haplotype relative risk study and association with clozapine response. *Molecular Psychiatry*.

[11] Malhotra AK, Goldman D, Mazzanti C, Clifton A, Breier A, Pickar D (1998). A functional serotonin transporter (5-HTT) polymorphism is associated with psychosis in neuroleptic-free schizophrenics. *Molecular Psychiatry*.

[12] Malhotra AK, Goldman D, Ozaki N, Breier A, Buchanan R, Pickar D (1996). Lack of association between polymorphisms in the 5-HT(2A) receptor gene and the antipsychotic response to clozapine. *The American Journal of Psychiatry*.

[13] Masellis M, Basile V, Meltzer HY (1998). Serotonin subtype 2 receptor genes and clinical response to clozapine in schizophrenia patients. *Neuropsychopharmacology*.

[14] Arranz MJ, Munro J, Birkett J (2000). Pharmacogenetic prediction of clozapine response. *The Lancet*.

[15] Lee ST, Ryu S, Kim SR (2012). Association study of 27 annotated genes for clozapine pharmacogenetics: validation of preexisting studies and identification of a new candidate gene, ABCB1, for treatment response. *Journal of Clinical Psychopharmacology*.

[16] Schumacher J, Schulze TG, Wienker TF, Rietschel M, Nothen MM (2000). Pharmacogenetics of clozapine response. *The Lancet*.

[17] Shuldiner AR, O'Connell JR, Bliden KP (2009). Association of cytochrome P450 2C19 genotype with the antiplatelet effect and clinical efficacy of clopidogrel therapy. *Journal of the American Medical Association*.

[18] Link E, Parish S, Armitage J (2008). SLCO1B1 variants and statin-induced myopathy—a genomewide study. *The New England Journal of Medicine*.

[19] Cooper GM, Johnson JA, Langaee TY (2008). A genome-wide scan for common genetic variants with a large influence on warfarin maintenance dose. *Blood*.

[20] Cha PC, Mushiroda T, Takahashi A (2010). Genome-wide association study identifies genetic determinants of warfarin responsiveness for Japanese. *Human Molecular Genetics*.

[21] Takeuchi F, McGinnis R, Bourgeois S (2009). A genome-wide association study confirms *VKORC1*, *CYP2C9*, and *CYP4F2* as principal genetic determinants of warfarin dose. *PLoS Genetics*.

[22] Daly AK, Donaldson PT, Bhatnagar P (2009). *HLA-B^*∗*^5701* genotype is a major determinant of drug-induced liver injury due to flucloxacillin. *Nature Genetics*.

[23] Åberg K, Adkins DE, Bukszár J (2010). Genomewide association study of movement-related adverse antipsychotic effects. *Biological Psychiatry*.

[24] Adkins DE, Åberg K, McClay JL (2011). Genomewide pharmacogenomic study of metabolic side effects to antipsychotic drugs. *Molecular Psychiatry*.

[25] Adkins DE, Berg K, McClay JL (2010). A genomewide association study of citalopram response in major depressive disorder-a psychometric approach. *Biological Psychiatry*.

[26] Alkelai A, Greenbaum L, Rigbi A, Kanyas K, Lerer B (2009). Genome-wide association study of antipsychotic-induced parkinsonism severity among schizophrenia patients. *Psychopharmacology*.

[27] Lavedan C, Licamele L, Volpi S (2009). Association of the NPAS3 gene and five other loci with response to the antipsychotic iloperidone identified in a whole genome association study. *Molecular Psychiatry*.

[28] McClay JL, Adkins DE, Åberg K (2011). Genome-wide pharmacogenomic analysis of response to treatment with antipsychotics. *Molecular Psychiatry*.

[29] Volpi S, Heaton C, MacK K (2009). Whole genome association study identifies polymorphisms associated with QT prolongation during iloperidone treatment of schizophrenia. *Molecular Psychiatry*.

[30] Volpi S, Potkin SG, Malhotra AK, Licamele L, Lavedan C (2009). Applicability of a genetic signature for enhanced iloperidone efficacy in the treatment of schizophrenia. *Journal of Clinical Psychiatry*.

[31] Belmont JW, Hardenbol P, Willis TD (2003). The international HapMap project. *Nature*.

[32] Lucena MI, Molokhia M, Shen Y (2011). Susceptibility to amoxicillin-clavulanate-induced liver injury is influenced by multiple HLA class I and II alleles. *Gastroenterology*.

[33] Jablonski KA, McAteer JB, De Bakker PIW (2010). Common variants in 40 genes assessed for diabetes incidence and response to metformin and lifestyle intervention in the diabetes prevention program. *Diabetes*.

[34] Lubomirov R, Lulio JD, Fayet A (2010). ADME pharmacogenetics: investigation of the pharmacokinetics of the antiretroviral agent lopinavir coformuiated with ritonavir. *Pharmacogenetics and Genomics*.

[35] Suarez-Kurtz G, Vargens DD, Sortica VA, Hutz MH (2012). Accuracy of NAT2 SNP genotyping panels to infer acetylator phenotypes in African, Asian, Amerindian and admixed populations. *Pharmacogenomics*.

[36] Suarez-Kurtz G, Pena SD, Hutz MH (2012). Application of the *F*
_*ST*_ statistics to explore pharmacogenomic diversity in the Brazilian population. *Pharmacogenomics*.

[37] Gage BF, Eby C, Johnson JA (2008). Use of pharmacogenetic and clinical factors to predict the therapeutic dose of warfarin. *Clinical Pharmacology and Therapeutics*.

[38] Owen RP, Altman RB, Klein TE (2008). PharmGKB and the international warfarin pharmacogenetics consortium: the changing role for pharmacogenomic databases and single-drug pharmacogenetics. *Human Mutation*.

[39] Klein TE, Altman RB, Eriksson N (2009). Estimation of the warfarin dose with clinical and pharmacogenetic data. *The New England Journal of Medicine*.

[40] Shin J, Cao D (2011). Comparison of warfarin pharmacogenetic dosing algorithms in a racially diverse large cohort. *Pharmacogenomics*.

[41] McCormack M, Alfirevic A, Bourgeois S (2011). HLA-A^*∗*^3101 and carbamazepine-induced hypersensitivity reactions in Europeans. *The New England Journal of Medicine*.

[42] Chen P, Lin JJ, Lu CS (2011). Carbamazepine-induced toxic effects and HLA-B^*∗*^1502 screening in Taiwan. *The New England Journal of Medicine*.

[43] Yip VL, Marson AG, Jorgensen AL, Pirmohamed M, Alfirevic A (2012). HLA genotype and carbamazepine-induced cutaneous adverse drug reactions: a systematic review. *Clinical Pharmacology and Therapeutics*.

[44] Alfaro CL, Lam YWF, Simpson J, Ereshefsky L (1999). CYP2D6 status of extensive metabolizers after multiple-dose fluoxetine, fluvoxamine, paroxetine, or sertraline. *Journal of Clinical Psychopharmacology*.

[45] Alfaro CL, Lam YWF, Simpson J, Ereshefsky L (2000). CYP2D6 inhibition by fluoxetine, paroxetine, sertraline, and venlafaxine in a crossover study: intraindividual variability and plasma concentration correlations. *Journal of Clinical Pharmacology*.

[46] Robertson GR, Liddle C, Clarke SJ (2008). Inflammation and altered drug clearance in cancer: transcriptional repression of a human CYP3A4 transgene in tumor-bearing mice. *Clinical Pharmacology and Therapeutics*.

[47] Espnes KA, Heimdal KO, Spigset O (2012). A puzzling case of increased serum clozapine levels in a patient with inflammation and infection. *Therapeutic Drug Monitoring*.

[48] Stegmeier F, Warmuth M, Sellers WR, Dorsch M (2010). Targeted cancer therapies in the twenty-first century: lessons from imatinib. *Clinical Pharmacology and Therapeutics*.

[49] Gomez A, Ingelman-Sundberg M (2009). Pharmacoepigenetics: its role in interindividual differences in drug response. *Clinical Pharmacology and Therapeutics*.

[50] Baer-Dubowska W, Majchrzak-Celińska A, Cichocki M (2011). Pharmocoepigenetics: a new approach to predicting individual drug responses and targeting new drugs. *Pharmacological Reports*.

[51] Perera V, Gross AS, McLachlan AJ (2012). Influence of environmental and genetic factors on CYP1A2 activity in individuals of South Asian and European ancestry. *Clinical Pharmacology and Therapeutics*.

[52] Kacevska M, Ivanov M, Ingelman-Sundberg M (2012). Epigenomics and interindividual differences in drug response. *Clinical Pharmacology and Therapeutics*.

[53] Centers for Disease Control and Prevention

[54] Berg AO, Piper M, Armstrong K (2007). Recommendations from the EGAPP working group: testing for cytochrome P450 polymorphisms in adults with nonpsychotic depression treated with selective serotonin reuptake inhibitors. *Genetics in Medicine*.

[55] Becquemont L, Alfirevic A, Amstutz U (2011). Practical recommendations for pharmacogenomics-based prescription: 2010 ESF-UB conference on pharmacogenetics and pharmacogenomics. *Pharmacogenomics*.

[56] Crews KR, Gaedigk A, Dunnenberger HM (2012). Clinical Pharmacogenetics Implementation consortium (CPIC) guidelines for codeine therapy in the context of cytochrome P450 2D6 (CYP2D6) genotype. *Clinical Pharmacology and Therapeutics*.

[57] Fargher EA, Tricker K, Newman W (2007). Current use of pharmacogenetic testing: a national survey of thiopurine methyltransferase testing prior to azathioprine prescription. *Journal of Clinical Pharmacy and Therapeutics*.

[58] Johnson JA, Gong L, Whirl-Carrillo M (2011). Clinical Pharmacogenetics Implementation Consortium Guidelines for CYP2C9 and VKORC1 genotypes and warfarin dosing. *Clinical Pharmacology and Therapeutics*.

[59] Martin MA, Klein TE, Dong BJ, Pirmohamed M, Haas DW, Kroetz DL (2012). Clinical pharmacogenetics implementation consortium guidelines for hla-B genotype and abacavir dosing. *Clinical Pharmacology and Therapeutics*.

[60] Relling MV, Gardner EE, Sandborn WJ (2011). Clinical pharmacogenetics implementation consortium guidelines for thiopurine methyltransferase genotype and thiopurine dosing. *Clinical Pharmacology and Therapeutics*.

[61] Scott SA, Sangkuhl K, Gardner EE (2011). Clinical pharmacogenetics implementation consortium guidelines for cytochrome P450-2C19 (CYP2C19) genotype and clopidogrel therapy. *Clinical Pharmacology and Therapeutics*.

[62] Thompson MA, Aberg JA, Cahn P (2010). Antiretroviral treatment of adult HIV infection: 2010 recommendations of the International AIDS Society-USA panel. *Journal of the American Medical Association*.

[63] Wilke RA, Ramsey LB, Johnson SG (2012). The clinical pharmacogenomics implementation consortium: CPIC guideline for SLCO1B1 and simvastatin-induced myopathy. *Clinical Pharmacology and Therapeutics*.

[64] Chan SL, Suo C, Lee SC, Goh BC, Chia KS, Teo YY (2012). Translational aspects of genetic factors in the prediction of drug response variability: a case study of warfarin pharmacogenomics in a multi-ethnic cohort from Asia. *The Pharmacogenomics Journal*.

[65] Limdi NA, Wadelius M, Cavallari L (2010). Warfarin pharmacogenetics: a single VKORC1 polymorphism is predictive of dose across 3 racial groups. *Blood*.

[66] Altman RB (2011). Pharmacogenomics: “Noninferiority” is sufficient for initial implementation. *Clinical Pharmacology and Therapeutics*.

[67] Kelly CM, Pritchard KI (2012). *CYP2D6* genotype as a marker for benefit of adjuvant tamoxifen in postmenopausal women: lessons learned. *Journal of the National Cancer Institute*.

[68] Khoury MJ, Gwinn M, Dotson WD, Bowen MS (2011). Is there a need for PGxceptionalism?. *Genetics in Medicine*.

[69] Nakamura Y, Ratain MJ, Cox NJ, McLeod HL, Kroetz DL, Flockhart DA (2012). Re: CYP2D6 genotype and tamoxifen response in postmenopausal women with endocrine-responsive breast cancer: the breast International Group 1-98 trial. *Journal of the National Cancer Institute*.

[70] Woodcock J (2010). Assessing the clinical utility of diagnostics used in drug therapy. *Clinical Pharmacology and Therapeutics*.

[71] Lam YWF (2012). How much evidence is necessary for pharmacogenomic testing implementation?. *Clinical and Experimental Pharmacology*.

[72] Stingl JC, Brockmöller J (2011). Why, when, and how should pharmacogenetics be applied in clinical studies: current and future approaches to study designs. *Clinical Pharmacology and Therapeutics*.

[73] Karapetis CS, Khambata-Ford S, Jonker DJ (2008). K-ras mutations and benefit from cetuximab in advanced colorectal cancer. *The New England Journal of Medicine*.

[74] Deley MC, Ballman KV, Marandet J, Sargent D (2012). Taking the long view: how to design a series of phase III trials to maximize cumulative therapeutic benefit. *Clinical Trials*.

[75] Patterson SD, Cohen N, Karnoub M (2011). Prospective-retrospective biomarker analysis for regulatory consideration: white paper from the industry pharmacogenomics working group. *Pharmacogenomics*.

[76] Zhang JP, Lencz T, Malhotra AK (2010). D_2_ receptor genetic variation and clinical response to antipsychotic drug treatment: a meta-analysis. *The American Journal of Psychiatry*.

[77] Brass EP (2010). The gap between clinical trials and clinical practice: the use of pragmatic clinical trials to inform regulatory decision making. *Clinical Pharmacology and Therapeutics*.

[78] Tunis SR, Stryer DB, Clancy CM (2003). Practical clinical trials: increasing the value of clinical research for decision making in clinical and health policy. *Journal of the American Medical Association*.

[79] Anderson JL, Horne BD, Stevens SM (2012). A randomized and clinical effectiveness trial comparing two pharmacogenetic algorithms and standard care for individualizing warfarin dosing (CoumaGen-II). *Circulation*.

[80] Kirchheiner J, Bauer S, Meineke I (2002). Impact of CYP2C9 and CYP2C19 polymorphisms on tolbutamide kinetics and the insulin and glucose response in healthy volunteers. *Pharmacogenetics*.

[81] Mega JL, Simon T, Collet JP (2010). Reduced-function CYP2C19 genotype and risk of adverse clinical outcomes among patients treated with clopidogrel predominantly for PCI: a meta-analysis. *Journal of the American Medical Association*.

[82] Harmsze AM, van Werkum JW, Ten Berg JM (2010). CYP2C19^*∗*^2 and CYP2C9^*∗*^3 alleles are associated with stent thrombosis: a case-control study. *European Heart Journal*.

[83] Levine GN, Bates ER, Blankenship JC (2011). ACCF/AHA/SCAI guideline for percutaneous coronary intervention: executive summary: a report of the American College of Cardiology Foundation/American heart association task force on practice guidelines and the society for cardiovascular angiography and interventions. *Circulation*.

[84] Johnson JA, Roden DM, Lesko LJ, Ashley E, Klein TE, Shuldiner AR (2012). Clopidogrel: a case for indication-specific pharmacogenetics. *Clinical Pharmacology and Therapeutics*.

[85] Holmes DR, Dehmer GJ, Kaul S, Leifer D, O’Gara PT, Stein CM (2010). ACCF/AHA clinical alert: ACCF/AHA clopidogrel clinical alert: approaches to the FDA “boxed warning” a report of the American college of cardiology foundation task force on clinical expert consensus documents and the American heart association. *Circulation*.

[86] Lubomirov R, Colombo S, Di Iulio J (2011). Association of pharmacogenetic markers with premature discontinuation of first-line anti-HIV therapy: an observational cohort study. *Journal of Infectious Diseases*.

[87] Kauf TL, Farkouh RA, Earnshaw SR, Watson ME, Maroudas P, Chambers MG (2010). Economic efficiency of genetic screening to inform the use of abacavir sulfate in the treatment of HIV. *PharmacoEconomics*.

[88] Black WC (1990). The CE plane: a graphic representation of cost-effectiveness. *Medical Decision Making*.

[89] Rosenheck RA, Leslie DL, Doshi JA (2008). Second-generation antipsychotics: cost-effectiveness, policy options, and political decision making. *Psychiatric Services*.

[90] Sikich L, Frazier JA, McClellan J (2008). Double-blind comparison of first- and second-generation antipsychotics in early-onset schizophrenia and schizoaffective disorder: findings from the treatment of early-onset schizophrenia spectrum disorders (TEOSS) study. *American Journal of Psychiatry*.

[91] Lerer B, Segman RH, Fangerau H (2002). Pharmacogenetics of tardive dyskinesia: combined analysis of 780 patients supports association with dopamine D3 receptor gene Ser9Gly polymorphism. *Neuropsychopharmacology*.

[92] Bakker PR, van Harten PN, van Os J (2006). Antipsychotic-induced tardive dyskinesia and the Ser9Gly polymorphism in the DRD3 gene: a meta analysis. *Schizophrenia Research*.

[93] Furuta T, Shirai N, Kodaira M (2007). Pharmacogenomics-based tailored versus standard therapeutic regimen for eradication of H. pylori. *Clinical Pharmacology and Therapeutics*.

[94] Perlis RH, Ganz DA, Avorn J (2005). Pharmacogenetic testing in the clinical management of schizophrenia: a decision-analytic model. *Journal of Clinical Psychopharmacology*.

[95] Guzauskas GF, Hughes DA, Bradley SM, Veenstra DL (2012). A risk-benefit assessment of prasugrel, clopidogrel, and genotype-guided therapy in patients undergoing percutaneous coronary intervention. *Clinical Pharmacology and Therapeutics*.

[96] Reese ES, Daniel Mullins C, Beitelshees AL, Onukwugha E (2012). Cost-effectiveness of cytochrome P450 2C19 genotype screening for selection of antiplatelet therapy with clopidogrel or prasugrel. *Pharmacotherapy*.

[97] Eckman MH, Rosand J, Greenberg SM, Gage BF (2009). Cost-effectiveness of using pharmacogenetic information in warfarin dosing for patients with nonvalvular atrial fibrillation. *Annals of Internal Medicine*.

[98] You JHS, Tsui KKN, Wong RSM, Cheng G (2009). Potential clinical and economic outcomes of CYP2C9 and VKORC1 genotype-guided dosing in patients starting warfarin therapy. *Clinical Pharmacology and Therapeutics*.

[99] Frueh FW, Amur S, Mummaneni P (2008). Pharmacogenomic biomarker information in drug labels approved by the United States Food and Drug Administration: prevalence of related drug use. *Pharmacotherapy*.

[100] Hamburg MA, Collins FS (2010). The path to personalized medicine. *The New England Journal of Medicine*.

[101] Bartlett G, Zgheib N, Manamperi A (2012). Pharmacogenomics in primary care: a crucial entry point for global personalized medicine?. *Current Pharmacogenomics and Personalized Medicine*.

[102] Roberts JD, Wells GA, Le May MR (2012). Point-of-care genetic testing for personalisation of antiplatelet treatment (RAPID GENE): a prospective, randomised, proof-of-concept trial. *The Lancet*.

[103] Delaney JT, Ramirez AH, Bowton E (2012). Predicting clopidogrel response using DNA samples linked to an electronic health record. *Clinical Pharmacology and Therapeutics*.

[104] Schildcrout JS, Denny JC, Bowton E (2012). Optimizing drug outcomes through pharmacogenetics: a case for preemptive genotyping. *Clinical Pharmacology and Therapeutics*.

[105] Fernandez CA, Smith C, Yang W (2012). Concordance of DMET plus genotyping results with those of orthogonal genotyping methods. *Clinical Pharmacology and Therapeutics*.

[106] Kirchheiner J, Brøsen K, Dahl ML (2001). CYP2D6 and CYP2C19 genotype-based dose recommendations for antidepressants: a first step towards subpopulation-specific dosages. *Acta Psychiatrica Scandinavica*.

[107] Finkelman BS, Gage BF, Johnson JA, Brensinger CM, Kimmel SE (2011). Genetic warfarin dosing: tables versus algorithms. *Journal of the American College of Cardiology*.

[108] Stanek EJ, Sanders CL, Taber KA (2012). Adoption of pharmacogenomic testing by US physicians: results of a nationwide survey. *Clinical Pharmacology and Therapeutics*.

[109] Gurwitz D, Lunshof JE, Dedoussis G (2005). Pharmacogenomics education: International Society of pharmacogenomics recommendations for medical, pharmaceutical, and health schools deans of education. *The Pharmacogenomics Journal*.

[110] Lam YWF (2012). Rethinking pharmacogenomics education beyond health professionals: addressing the “Know-Do” gap across the personalized medicine innovation ecosystem. *Current Pharmacogenomics and Personalized Medicine*.

[111] Mccullough KB, Formea CM, Berg KD (2011). Assessment of the pharmacogenomics educational needs of pharmacists. *American Journal of Pharmaceutical Education*.

[112] Swen JJ, Nijenhuis M, De Boer A (2011). Pharmacogenetics: from bench to byte an update of guidelines. *Clinical Pharmacology and Therapeutics*.

[113] Overby CL, Tarczy-Hornoch P, Hoath JI, Kalet IJ, Veenstra DL (2010). Feasibility of incorporating genomic knowledge into electronic medical records for pharmacogenomic clinical decision support. *BMC Bioinformatics*.

[114] Hicks JK, Crews KR, Hoffman JM (2012). A clinician-driven automated system for integration of pharmacogenetic interpretations into an electronic medical record. *Clinical Pharmacology and Therapeutics*.

[115] McCarty CA, Nair A, Austin DM, Giampietro PF (2006). Informed consent and subject motivation to participate in a large, population-based genomics study: the marshfield clinic personalized medicine research project. *Community Genetics*.

[116] Xu H, Stenner SP, Doan S, Johnson KB, Waitman LR, Denny JC (2010). MedEx: a medication information extraction system for clinical narratives. *Journal of the American Medical Informatics Association*.

[117] Kho AN, Pacheco JA, Peissig PL (2011). Electronic medical records for genetic research: results of the eMERGE consortium. *Science Translational Medicine*.

[118] Ramirez AH, Shi Y, Schildcrout JS (2012). Predicting warfarin dosage in European-Americans and African-Americans using DNA samples linked to an electronic health record. *Pharmacogenomics*.

[119] Jha AK, Desroches CM, Campbell EG (2009). Use of electronic health records in U.S. hospitals. *The New England Journal of Medicine*.

[120] Cohen J, Wilson A, Manzolillo K (2012). Clinical and economic challenges facing pharmacogenomics. *The Pharmacogenomics Journal*.

[121] Trosman JR, Van Bebber SL, Phillips KA (2011). Health technology assessment and private payers's coverage of personalized medicine. *The American Journal of Managed Care*.

[122] Food Drug Administration Guidance on pharmacogenetic tests and genetic tests for heritable markers.

[123] Elkin EB, Weinstein MC, Winer EP, Kuntz KM, Schnitt SJ, Weeks JC (2004). HER-2 testing and trastuzumab therapy for metastatic breast cancer: a cost-effectiveness analysis. *Journal of Clinical Oncology*.

[124] Van den Akker-Van ME, Gurwitz D, Detmar SB (2006). Cost-effectiveness of pharmacogenomics in clinical practice: a case study of thiopurine methyltransferase genotyping in acute lymphoblastic leukemia in Europe. *Pharmacogenomics*.

[125] Hughes AR, Spreen WR, Mosteller M (2008). Pharmacogenetics of hypersensitivity to abacavir: from PGx hypothesis to confirmation to clinical utility. *The Pharmacogenomics Journal*.

[126] Food US, Drug Administration FDA approves Xalkori with companion diagnostic for a type of late-stage lung cancer.

[127] Blair ED (2008). Assessing the value-adding impact of diagnostic-type tests on drug development and marketing. *Molecular Diagnosis and Therapy*.

[128] Epstein RS, Moyer TP, Aubert RE (2010). Warfarin genotyping reduces hospitalization rates results from the MM-WES (Medco-Mayo Warfarin Effectiveness study). *Journal of the American College of Cardiology*.

[129] Bevan JL, Lynch JA, Dubriwny TN (2003). Informed lay preferences for delivery of racially varied pharmacogenomics. *Genetics in Medicine*.

[130] Haga SB, O’Daniel JM, Tindall GM, Lipkus IR, Agans R (2011). Survey of US public attitudes toward pharmacogenetic testing. *The Pharmacogenomics Journal*.

[131] Loukides G, Gkoulalas-Divanis A, Malin B (2010). Anonymization of electronic medical records for validating genome-wide association studies. *Proceedings of the National Academy of Sciences of the United States of America*.

[132] Aberdeen J, Bayer S, Yeniterzi R (2010). The MITRE identification scrubber toolkit: design, training, and assessment. *International Journal of Medical Informatics*.

[133] Dressler LG, Terry SF (2009). How will GINA influence participation in pharmacogenomics research and clinical testing?. *Clinical Pharmacology and Therapeutics*.

[134] Janssens ACJW, Gwinn M, Bradley LA, Oostra BA, van Duijn CM, Khoury MJ (2008). A critical appraisal of the scientific basis of commercial genomic profiles used to assess health risks and personalize health interventions. *American Journal of Human Genetics*.

[135] Spencer DH, Lockwood C, Topol E (2011). Direct-to-consumer genetic testing: reliable or risky?. *Clinical Chemistry*.

[136] Ashley EA, Butte AJ, Wheeler MT (2010). Clinical assessment incorporating a personal genome. *The Lancet*.

[137] Grecco N, Cohen N, Warner AW (2012). PhRMA survey of pharmacogenomic and pharmacodynamic evaluations: what next?. *Clinical Pharmacology and Therapeutics*.

[138] (2011). *Strattera*.

[139] Inoue A, Suzuki T, Fukuhara T (2006). Prospective phase II study of gefitinib for chemotherapy-naïve patients with advanced non-small-cell lung cancer with epidermal growth factor receptor gene mutations. *Journal of Clinical Oncology*.

[140] Sequist LV, Martins RG, Spigel D (2008). First-line gefitinib in patients with advanced non-small-cell lung cancer harboring somatic EGFR mutations. *Journal of Clinical Oncology*.

[141] Tamura K, Okamoto I, Kashii T (2008). Multicentre prospective phase II trial of gefitinib for advanced non-small cell lung cancer with epidermal growth factor receptor mutations: results of the west Japan thoracic oncology group trial (WJTOG0403). *British Journal of Cancer*.

[142] Yang CH, Yu CJ, Shih JY (2008). Specific EGFR mutations predict treatment outcome of stage IIIB/IV patients with chemotherapy-naive non-small-cell lung cancer receiving first-line gefitinib monotherapy. *Journal of Clinical Oncology*.

[143] Kobayashi K, Inoue A, Usui K (2009). First-line gefitinib for patients with advanced non-small-cell lung cancer harboring epidermal growth factor receptor mutations without indication for chemotherapy. *Journal of Clinical Oncology*.

[144] Singer JB, Lewitzky S, Leroy E (2010). A genome-wide study identifies HLA alleles associated with lumiracoxib-related liver injury. *Nature Genetics*.

[145] Spraggs CF, Parham LR, Hunt CM, Dollery CT (2012). Lapatinib-induced liver injury characterized by class II HLA and Gilbert's syndrome genotypes. *Clinical Pharmacology and Therapeutics*.

[146] Surh LC, Pacanowski MA, Haga SB (2010). Learning from product labels and label changes: how to build pharmacogenomics into drug-development programs. *Pharmacogenomics*.

